# Predicting Critical Outcomes in Suspected Cardiopulmonary Emergencies Using Dispatch Narratives: Temporal Validation Study

**DOI:** 10.2196/97672

**Published:** 2026-08-14

**Authors:** Zhe Li, Lei Shi, Chunting Luo, Siqi Huang, Jianmin Qin, Min Yao, Sanshan Zhu, Zhengzhuang Huang, Yinghua Nong, Guozheng Qiu, Liwen Lyu

**Affiliations:** 1 The People's Hospital of Guangxi Zhuang Autonomous Region, No. 6 Taoyuan Road, Nanning, Guangxi, 530021, China, +86 15978194424; 2 Nanning Emergency Medical Center, Nanning, Guangxi, China

**Keywords:** digital health, emergency medical dispatch, natural language processing, machine learning, clinical decision support, prehospital care, risk stratification, artificial intelligence

## Abstract

**Background:**

Early risk stratification in emergency medical services (EMS) is essential for patients presenting with acute cardiopulmonary symptoms, yet prehospital decision-making at the dispatch stage is often based on limited structured information. Free-text dispatch narratives may contain additional clinical signals, but their role in early risk assessment remains insufficiently characterized.

**Objective:**

This study aims to develop and temporally validate a natural language processing–assisted machine learning framework for early risk stratification using free-text EMS dispatch narratives and to evaluate its incremental value beyond conventional structured dispatch information.

**Methods:**

We conducted a population-based retrospective cohort study using EMS dispatch records from Nanning, China, between 2021 and 2025. Adult patients with suspected cardiopulmonary symptoms were identified based on predefined complaint keywords. After excluding nonmedical and incomplete records, 38,523 cases with available free-text narratives were included. To simulate real-world deployment, data from 2021 to 2024 (n=28,332) were used for model development, and 2025 data (n=10,191) served as an independent temporal test cohort. Dispatch narratives were processed using a natural language processing pipeline based on character-level n-grams and combined with structured variables (age, sex, call time) in a multimodal machine learning framework. The primary outcome was a composite prehospital critical outcome comprising death, clinical deterioration, or lack of response to initial treatment. Model performance was evaluated using area under the receiver operating characteristic curve (AUROC), area under the precision-recall curve, calibration, and decision curve analysis.

**Results:**

Among the 38,523 included patients, 12,476 (32.4%) experienced the primary composite outcome. The median age was 68.0 (IQR 54.0‐79.0) years, and 23,019 (59.8%) patients were male. In the temporally independent 2025 cohort, an expanded structured baseline model achieved an AUROC of 0.681 (95% CI 0.669‐0.693). Incorporation of narrative features improved performance (AUROC 0.803, 95% CI 0.792‐0.814), with marginal additional gain from multimodal integration (AUROC 0.808, 95% CI 0.798‐0.818). The multimodal model achieved an area under the precision-recall curve of 0.630 (95% CI 0.611‐0.651), substantially exceeding the no-skill baseline defined by the outcome prevalence in the temporal test cohort (2464/10,191, 24.2%). Model performance remained consistent across age and sex subgroups, and calibration was acceptable (Brier score 0.1878). In a risk enrichment analysis, the top 10% (n=1019) of predicted high-risk cases accounted for 32.1% (n=791) of all critical outcomes, representing a 3.2-fold enrichment. Decision curve analysis indicated a higher net benefit compared with treat-all and treat-none strategies across a range of threshold probabilities.

**Conclusions:**

Free-text dispatch narratives contain clinically relevant information associated with early risk stratification in patients with suspected cardiopulmonary emergencies. Incorporating narrative-derived features into a structured modeling framework may complement existing EMS dispatch systems and support more informed decision-making prior to patient contact. Further external validation and prospective evaluation are warranted.

## Introduction

Acute cardiopulmonary emergencies represent one of the most time-critical categories encountered by emergency medical services (EMS), frequently presenting with symptoms such as chest pain, dyspnea, syncope, or sudden loss of consciousness [1]. These conditions—including acute coronary syndromes, malignant arrhythmias, and cardiogenic shock—are associated with rapid clinical deterioration and high mortality if not promptly recognized and managed [2]. For EMS teams, the period before patient contact is particularly crucial. Decisions made at the time of dispatch—such as mobilizing advanced life support (ALS) resources, preparing defibrillation or airway equipment, and predetermining the most appropriate destination hospital—can significantly influence the timeliness and effectiveness of subsequent care [3]. In this context, early risk stratification based solely on information obtained during the emergency call has the potential to optimize prehospital readiness and reduce delays in definitive treatment [4].

In current practice, early assessment of patients with suspected acute cardiovascular conditions is largely indirect and experience-driven [5]. Before arriving on scene, EMS personnel and dispatchers must rely on limited structured information, such as patient age, reported symptoms, and predefined chief complaint categories, to infer severity. However, such structured variables often lack granularity and fail to reflect the dynamic and heterogeneous nature of cardiovascular emergencies [6]. As a result, prehospital decision-making frequently depends on subjective interpretation rather than standardized, quantitative risk assessment. This reliance on experience may lead to variability in triage decisions, with potential consequences including delayed escalation of care for high-risk patients or unnecessary allocation of advanced resources to lower-risk cases [7]. These challenges underscore a critical gap in current dispatch systems: the absence of objective tools capable of translating early call information into actionable risk stratification [8].

During emergency calls, particularly in cases involving cardiovascular complaints, callers often provide detailed narrative descriptions that extend beyond structured categories [9]. Expressions such as “no response,” “sudden collapse,” “gasping,” or “progressively worsening chest discomfort” may convey important clinical cues related to hemodynamic instability or impending cardiac arrest [10]. These free-text narratives, whether documented by dispatchers or generated through real-time transcription systems, contain rich contextual information that is not captured in conventional triage frameworks [11]. With the rapid development of natural language processing (NLP) techniques, it has become increasingly feasible to systematically analyze such unstructured data and extract clinically meaningful patterns [12,13]. However, many previous clinical NLP studies have focused on longer clinical narratives or electronic health records, while text-based analysis of ultrashort, time-sensitive dispatch communications remains limited. Moreover, previous EMS NLP models have primarily targeted single high-acuity conditions, such as out-of-hospital cardiac arrest. In contrast, comprehensive early risk stratification across heterogeneous cardiopulmonary presentations encountered at dispatch remains largely underexplored. Crucially, unlike models that rely on prehospital vital signs or clinician assessments after EMS arrival, our framework performs risk stratification using unstructured information available at the time of the emergency call, enabling decision support at the earliest stage of the EMS pathway—prior to patient contact. Finally, because previous machine learning models in EMS triage frequently rely on random data splits that may overestimate performance, the generalizability of these approaches under real-world temporal variation has not been fully established [14]. Therefore, this study advances prior work by developing a lightweight, interpretable NLP framework for emergency dispatch and evaluating its real-world robustness through a rigorous temporal validation strategy.

In light of these considerations, rather than focusing on algorithmic innovation, the present study aimed to develop and temporally validate an automated risk stratification framework for patients presenting with cardiovascular-related complaints during emergency calls, with a primary focus on evaluating its real-world clinical applicability. Leveraging a large real-world EMS dataset, we applied NLP methods to extract features from dispatch narratives and integrated them with structured variables within a multimodal machine learning framework. Model performance was evaluated using a temporally independent test cohort to assess robustness under evolving case mix and usage patterns. In addition, we examined model interpretability and clinical usefulness through feature attribution and risk enrichment analyses. By focusing on early-stage information available prior to patient contact, this study seeks to explore the potential of NLP-assisted models to support prehospital decision-making and optimize resource allocation in time-sensitive cardiopulmonary emergencies.

## Methods

### Study Design and Data Source

This study was designed as a population-based, real-world retrospective cohort analysis using data derived from the EMS system in Nanning, Guangxi Zhuang Autonomous Region, China. All emergency calls are centrally coordinated through the Nanning Emergency Medical Center, which operates the unified municipal dispatch system and provides prehospital emergency care across both urban and surrounding suburban areas.

The EMS database integrates dispatch information with standardized electronic prehospital medical records completed by on-scene physicians, allowing linkage between call-level information and clinical outcomes. This structure enabled comprehensive capture of both structured variables and free-text narratives generated during the dispatch process and subsequent patient assessment.

### Study Population and Temporal Validation Strategy

All EMS dispatch records between January 1, 2021, and December 31, 2025, were screened. Patients were eligible for inclusion if the emergency call was associated with symptoms suggestive of acute cardiopulmonary conditions, identified through predefined dispatch complaint categories and keyword-based filtering of call narratives (based on standard EMS dispatch guidelines). These included presentations such as chest pain, dyspnea, syncope, and altered consciousness. We excluded records related to trauma, nonmedical events, interfacility transfers, and cases with missing key variables, including dispatch narratives or outcome labels. Given the extremely low rate of missing data (<1%), a complete case analysis approach was used. After applying these criteria, a total of 38,523 cases were included in the final analysis.

To simulate real-world prospective deployment and minimize information leakage, a temporal validation strategy was adopted. Data from January 2021 to December 2024 were used as the training cohort (n=28,332) for model development and internal validation, while data from January to December 2025 were reserved as an independent temporal test cohort (n=10,191). This approach allowed the evaluation of model performance under natural variations in case mix and EMS utilization over time.

### Definition of the Clinical Outcome

The primary outcome of interest was a composite end point uniformly defined as a “critical outcome.” This label was derived from standardized on-scene medical records completed by EMS physicians and reflected patients experiencing a critical outcome requiring urgent intervention. Operationally, these outcomes were recorded by attending EMS physicians using electronic prehospital care reports immediately following the mission. Data standardization was enforced by municipal EMS protocols, and validity was supported by routine electronic logic checks and administrative quality assurance within the EMS database. To improve objectivity and reproducibility, the composite end point was anchored to operational EMS records, including documented on-scene death, requirement for ALS interventions (eg, airway management, cardiopulmonary resuscitation), or persistent instability necessitating immediate emergency department resuscitation upon hospital arrival. These criteria were based on structured EMS documentation fields to reduce interphysician variability.

Specifically, outcome labels were generated using predefined operational criteria and determined using standardized structured fields in the prehospital electronic medical record rather than subjective free-text descriptions, thereby improving interrater consistency. Critical outcomes included (1) death at the scene, (2) persistent hemodynamic or respiratory instability (eg, systolic blood pressure persistently <90 mm Hg requiring vasopressors, or peripheral capillary oxygen saturation [SpO2] <90% despite high-flow oxygen) despite initial resuscitative efforts, and (3) refractory or rapidly worsening clinical status necessitating immediate escalation of care. This composite end point was chosen to capture clinically meaningful high-risk cases that would benefit from early identification and optimized prehospital preparation. Although not formally validated as a standardized end point, this composite outcome reflects the occurrence of operationally relevant critical outcomes and has been widely adopted in EMS-based risk stratification studies focusing on early decision-making.

### NLP Pipeline

To prevent information leakage, the dataset was temporally partitioned into training (2021‐2024) and test (2025) cohorts before vocabulary construction, feature selection, and model development. Free-text data, specifically the dispatch narratives documented by call-takers during the emergency call, were processed using a structured NLP pipeline. Prior to feature extraction, all text entries underwent standardized preprocessing, including removal of punctuation, special characters, and noninformative stop-words (using a customized Chinese medical stop-word dictionary), to reduce noise and improve signal consistency. Crucially, the NLP pipeline, including vocabulary construction and selection of the top 300 most frequent n-grams, was developed exclusively using the training cohort. The independent temporal test cohort remained isolated throughout model development and was subsequently transformed using only the predefined vocabulary and preprocessing rules derived from the training data. No information from the temporal test cohort was used during feature engineering, model training, or hyperparameter tuning.

The cleaned text was then transformed into character-level n-grams, specifically bigrams, to capture local semantic patterns within short phrases commonly used in emergency communication. Given that the dispatch narratives were recorded in Chinese, this character-level tokenization strategy was deliberately used. It effectively bypasses the inaccuracies commonly associated with Chinese word segmentation tools when parsing highly colloquial, fragmented, or abbreviated emergency medical texts, allowing for direct capture of native semantic structures. To ensure computational efficiency and reduce dimensionality, feature selection was performed based on term frequency across the training corpus. The top 300 most frequent n-grams were retained as the core feature set. These selected features were subsequently encoded into a structured numerical matrix. Given the ultra-short nature of emergency dispatch narratives, where term recurrence within a single record is exceedingly rare, a binary encoding scheme (presence vs absence) was used. In this specific short-text context, binary encoding effectively captures the essential semantic signal (functioning as a Boolean term frequency) without the added complexity of global weighting schemes such as term frequency-inverse document frequency (TF-IDF).

While advanced deep learning architectures such as transformer-based large language models (eg, bidirectional encoder representations from transformers, BERT) are increasingly used in medical informatics, a lightweight, explainable, n-gram-based approach was deliberately selected for this study. Dispatch narratives are inherently highly fragmented, abbreviated, and lack complex grammatical structures, rendering the contextual advantages of deep learning models marginal. Furthermore, the explicit n-gram matrix provides greater semantic transparency, which enhances the interpretability of downstream machine learning predictions—a mandatory prerequisite for building algorithmic trust in high-stakes emergency triage. Importantly, feature selection (top 300 n-grams) was performed exclusively within the training dataset to prevent information leakage into the temporal test cohort.

### Machine Learning Development and Multimodal Fusion

To leverage both structured and unstructured information, a multimodal modeling framework was constructed by combining the NLP-derived features with baseline structured variables, including age, sex, and call time. Given the class imbalance in the training cohort, with a relatively lower proportion of critical outcomes, class weighting was incorporated into the model training process to enhance sensitivity toward high-risk cases. Specifically, the scale_pos_weight hyperparameter in the Extreme Gradient Boosting (XGBoost) algorithm was defined as the ratio of negative (noncritical) to positive (critical outcome) in the training set.

The primary model was developed using XGBoost, selected for its ability to handle nonlinear relationships and interactions. Hyperparameter tuning (including parameters such as maximum depth, learning rate, and subsample ratio) was conducted via grid search using 5-fold cross-validation exclusively within the training cohort to optimize the final model parameters. For benchmarking purposes, additional models, including random forest and penalized logistic regression, were also trained under the same framework. To ensure a fair comparison and reproducibility, all model hyperparameters were optimized within the training set only, without access to the temporal test cohort.

### Model Evaluation, Explainability, and Clinical Usefulness

Model performance was evaluated on the independent 2025 temporal test cohort. Discrimination was assessed using the area under the receiver operating characteristic curve (AUROC). Given the class imbalance, additional metrics, including the area under the precision-recall curve (AUPRC) and *F*_1_-score, were calculated to provide a more comprehensive assessment of model performance. Calibration was evaluated using the Brier score.

To enhance interpretability, Shapley Additive Explanations (SHAP) were applied to quantify the contribution of individual features to model predictions, allowing the identification of key textual patterns associated with increased risk.

To assess potential clinical applicability, a risk enrichment analysis was performed by ranking patients according to predicted risk and evaluating the proportion of true critical outcomes captured within predefined high-risk strata (top 5%, 10%, and 20%). In addition, decision curve analysis (DCA) was conducted to estimate the net benefit across a range of threshold probabilities, comparing the model against default strategies of treating all or no patients as high risk.

### Statistical Analysis

Continuous variables were presented as medians with IQRs and compared using the Mann-Whitney *U* test, given their nonnormal distributions. Categorical variables were summarized as counts (percentages) and compared using the Pearson chi-square test or Fisher exact test, as appropriate.

For model performance evaluation, 95% CIs for the AUROC were computed. Statistical comparisons of discriminative performance between the baseline structured model, the NLP-only model, and the multimodal fusion model were conducted using the DeLong test. Given the inherent class imbalance in critical outcomes, the AUPRC and *F*_1_-scores were additionally calculated as robust evaluation metrics. The clinical usefulness of the predictive models was assessed using DCA to quantify the net benefit across a range of threshold probabilities.

All data preprocessing, machine learning development, and statistical analyses were performed using R software (version 4.3.2; R Foundation for Statistical Computing). Key R packages used included xgboost for model training, pROC for ROC analysis and DeLong tests, dcurves for DCA, and relevant libraries for SHAP value computation and visualization. A 2-sided *P* value <.05 was considered statistically significant.

### Ethical Considerations

This study was approved by the Institutional Review Board of Guangxi Zhuang Autonomous Region People’s Hospital (approval number KY-KJT-2024‐239). The requirement for informed consent was waived because of the retrospective study design and the use of anonymized EMS data.

Additional methodological details and model specifications are provided in Multimedia Appendix 1. The completed TRIPOD (Transparent Reporting of a Multivariable Prediction Model for Individual Prognosis or Diagnosis) reporting checklist is available in Checklist 1.

## Results

### Study Population and Temporal Dataset Shift

**Figure 1.**
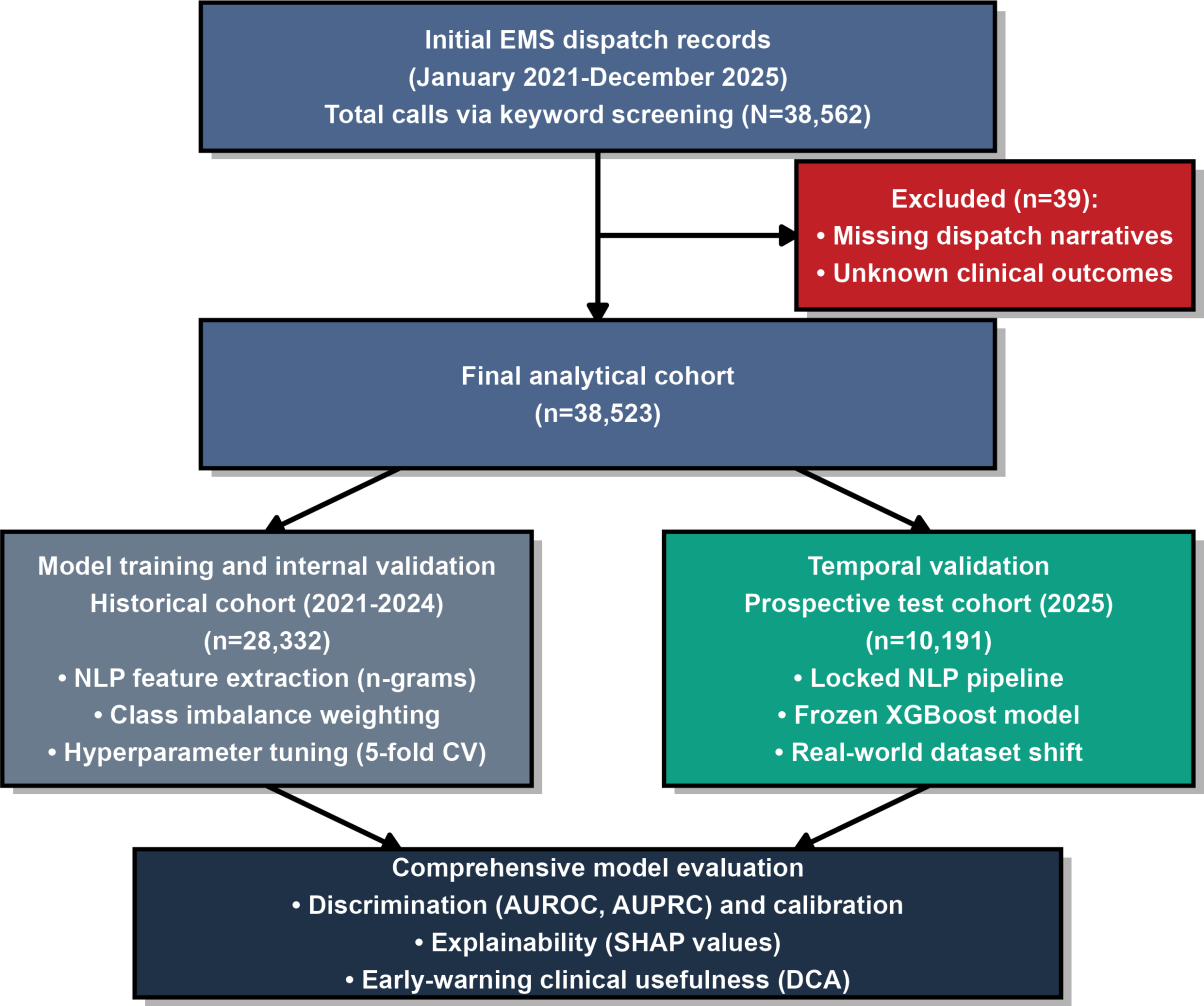
Study flowchart and temporal validation design. AUPRC: area under the precision-recall curve; AUROC: area under the receiver operating characteristic curve; CV: cross-validation; DCA: decision curve analysis; EMS: emergency medical services; NLP: natural language processing; SHAP: Shapley Additive Explanations; XGBoost: Extreme Gradient Boosting.

A total of 38,523 EMS dispatch records with available free-text narratives were included in the final analytic cohort. Historical data from 2021 to 2024 (n=28,332) were used for model development, while an independent temporal cohort from 2025 (n=10,191) served as the temporal validation dataset (Figure 1).

The flow diagram illustrates the patient selection process and the dual-track methodological framework. From an initial pool of 38,562 dispatch records screened via cardiopulmonary symptom keywords, 38,523 eligible cases were included after excluding records with missing dispatch narratives or unknown clinical outcomes. To simulate real-world prospective deployment and prevent data leakage, the cohort was temporally split into a historical training dataset (2021‐2024, n=28,332) and an independent temporal test dataset (2025, n=10,191). The diagram outlines the NLP pipeline, machine learning development, and comprehensive model evaluation strategies.

Baseline demographic characteristics were highly comparable between the 2 cohorts (Table 1). The median patient age was 68.0 (IQR 54.0‐79.0; *P*=.52) years in both groups, and the proportion of male patients was similar (n=16,872, 59.6% vs n=6147, 60.3%; *P*=.18). The temporal distribution of EMS calls was also consistent, with a median call hour of 13:00 (IQR 8.0-18.0) in both cohorts (*P*=.14). Structured major complaint categories, including dyspnea (n=10,265, 36.2% vs n=3599, 35.3%; *P*=.11), syncope (n=6692, 23.6% vs n=2367, 23.2%; *P*=.44), chest tightness (n=5082, 17.9% vs n=1877, 18.4%; *P*=.27), and chest pain (n=2387, 8.4% vs n=894, 8.8%; *P*=.28), showed stable distributions across the 2 periods.

**Figure 2.**
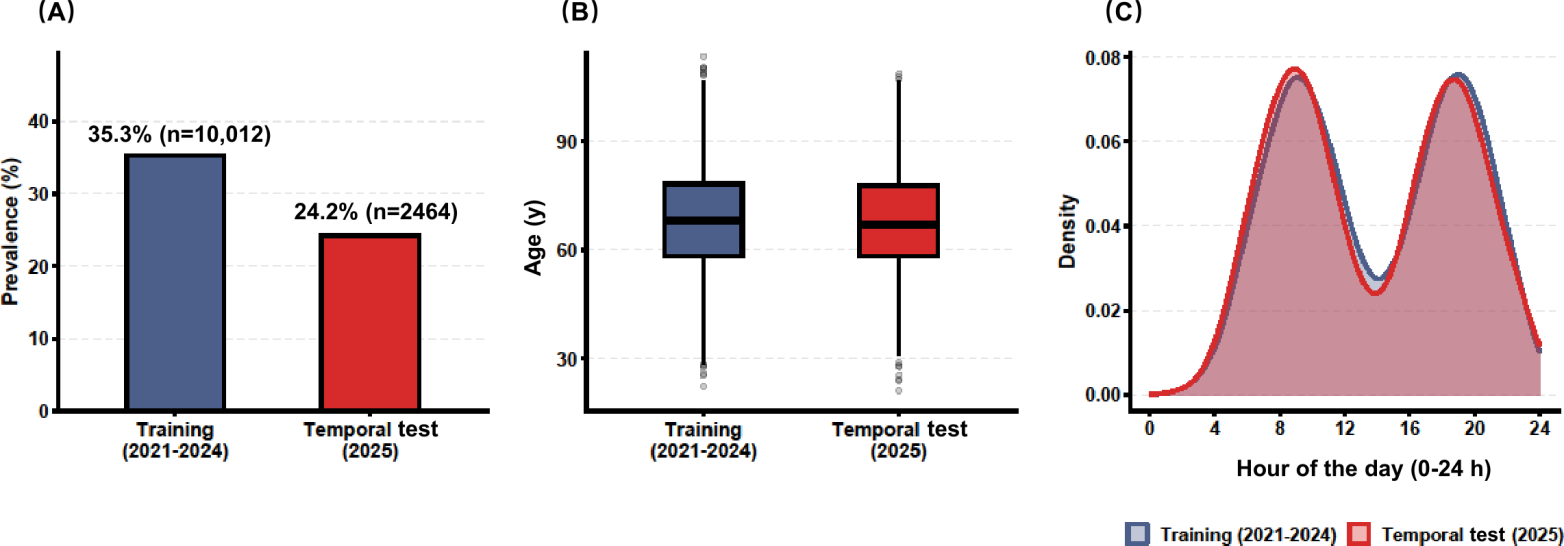
Temporal dataset shift and baseline stability between the historical training and prospective validation cohorts. (A) Bar chart demonstrating a significant decrease in the prevalence of critical outcomes from 35.3% (n=10,012) in the 2021‐2024 training cohort to 24.2% (n=2464) in the 2025 test cohort (*P*<.001), reflecting a natural dataset shift in real-world emergency medical services (EMS) use. (B) Box plots illustrating the stable distribution of patient age across the 2 cohorts (median 68.0, IQR 54.0‐79.0 y, *P*=.52). (C) Density plots showing highly consistent bimodal circadian rhythms in emergency call hours between the training and test cohorts (*P*=.14).

However, temporal dataset shifts were observed in the underlying clinical severity. The prevalence of critical outcomes decreased from 35.3% (n=10,012) in the historical training cohort to 24.2% (n=2464) in the 2025 temporal validation cohort (*P*<.001; Figure 2). Furthermore, the prevalence of certain NLP-extracted latent semantic signatures, such as second-hand reporting (n=11,070, 39.1% vs n=4199, 41.2%; *P*<.001) and convulsions (n=9129, 32.2% vs n=3651, 35.8%; *P*<.001), exhibited significant temporal fluctuations. The presence of this natural dataset shift provided a stringent real-world environment for evaluating model robustness under changing clinical distributions.

**Table 1.** Baseline characteristics, structured complaints, and natural language processing (NLP)–extracted signatures across temporal cohorts^a^.

Variables^b^	Overall cohort (N=38,523)	Training cohort (2021‐2024) (n=28,332)	Temporal test cohort (2025) (n=10,191)	*P* value
Demographics and call metrics				
Age (y), median (IQR)	68.0 (54.0‐79.0)	68.0 (54.0‐79.0)	68.0 (53.0‐79.0)	.52
Male sex, n (%)	23,019 (59.8)	16,872 (59.6)	6147 (60.3)	.18
Call hour, median (IQR)	13.0 (8.0‐18.0)	13.0 (8.0‐18.0)	13.0 (8.0‐18.0)	.14
Structured complaint category, n (%)				
Dyspnea or breathing difficulty	13,864 (36.0)	10,265 (36.2)	3599 (35.3)	.19
Syncope or unconsciousness	9059 (23.5)	6692 (23.6)	2367 (23.2)	.44
Chest tightness	6959 (18.1)	5082 (17.9)	1877 (18.4)	.28
Chest pain or angina	3281 (8.5)	2387 (8.4)	894 (8.8)	.28
NLP-extracted high-risk signatures, n (%)				
Second-hand reporting (bystander)	15,269 (39.6)	11,070 (39.1)	4199 (41.2)	<.001
Convulsions or seizure-like movements	12,780 (33.2)	9129 (32.2)	3651 (35.8)	<.001
Coma or altered mental status	8306 (21.6)	6033 (21.3)	2273 (22.3)	.04
Unresponsive to verbal stimuli	7897 (20.5)	5785 (20.4)	2112 (20.7)	.52
Diaphoresis or cold sweat	3965 (10.3)	2889 (10.2)	1076 (10.6)	.31
Clinical outcome				
Critical outcome, n (%)	12,476 (32.4)	10,012 (35.3)	2464 (24.2)	<.001

a Categorical variables are presented as counts (percentages) and compared using the Pearson *χ*2 test. While standard demographics and complaint categories remained stable, temporal dataset shifts were observed in the prevalence of critical outcomes and latent semantic signatures (eg, second-hand reporting, convulsions) in the 2025 test cohort. Percentages for categories do not sum to 100% due to multiple concurrent symptoms.

b Continuous variables are presented as median (IQR) and compared using the Mann-Whitney *U* test.

### Incremental Value of Dispatch Narratives

**Figure 3.**
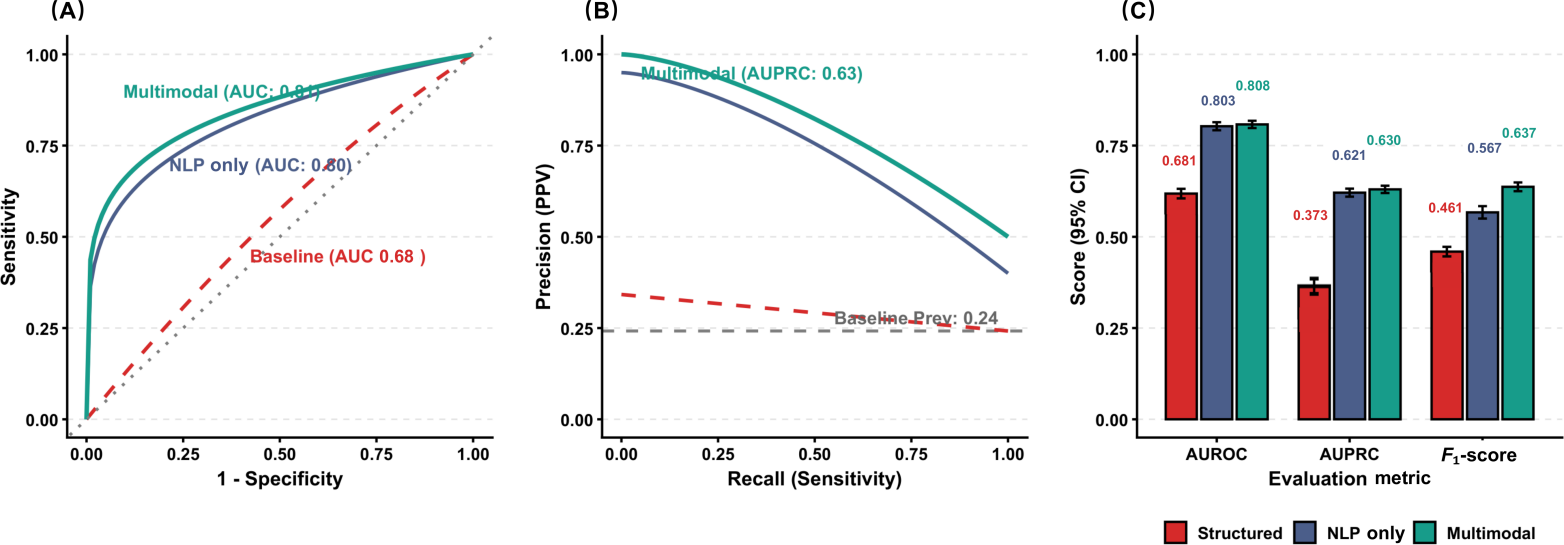
Incremental predictive value of unstructured dispatch narratives. (A) Receiver operating characteristic (ROC) curves comparing the discriminative performance of the expanded structured baseline model (incorporating age, sex, call hour, and complaint categories; red dashed line), and the multimodal fusion model (teal thick line) on the 2025 temporal test cohort. (B) Precision-recall (PR) curves for the 3 models, evaluating performance under class imbalance. The horizontal dashed line represents the baseline prevalence of critical outcomes (0.242). The multimodal model achieved the highest area under the precision-recall curve (AUPRC; 0.630). (C) Bar plot summarizing the area under the receiver operating characteristic curve (AUROC), AUPRC, and *F*_1_-score across the 3 models. Error bars indicate 95% CIs, demonstrating the meaningful incremental prognostic value provided by free-text dispatch narratives. AUC: area under the curve; NLP: natural language processing.

Structured triage variables alone demonstrated improved but still limited predictive ability for identifying critical outcomes in the temporal validation cohort. An expanded structured baseline model incorporating age, sex, call hour, and routinely available structured complaint categories (dyspnea, syncope, chest pain, and chest tightness) achieved an AUROC of 0.681 (95% CI 0.669‐0.693; Table 2). In contrast, models incorporating unstructured dispatch narratives substantially improved predictive discrimination beyond the expanded structured baseline model. The NLP-only model achieved an AUROC of 0.803 (95% CI 0.792‐0.814). The best performance was observed in the multimodal model integrating both structured variables and NLP-derived textual features, which achieved an AUROC of 0.808 (95% CI 0.798‐0.818; Table 2, Figure 3A).

Given the outcome prevalence of 24.2% in the temporal test cohort, precision-recall analysis was additionally performed to assess model performance under class imbalance. The multimodal model achieved an AUPRC of 0.630 (95% CI 0.611‐0.651), markedly exceeding the baseline prevalence of 0.242 (Figure 3B). Across classification metrics, the multimodal model consistently outperformed other approaches, achieving the highest *F*_1_-score (0.637) and an improved positive predictive value (PPV 0.571) compared with the expanded structured baseline model (PPV 0.341) (Table 2, Figure 3C).

**Table 2.** Incremental predictive value of automated triage models on the 2025 temporal test cohort^a^.

Model or features included	AUROC^b^ (95% CI)	AUPRC^c^	Sensitivity	Specificity	PPV^d^	NPV^e^	*F*_1_-score
Model A: expanded structured baseline (age, sex, call hour, and complaint categories)	0.681 (0.669‐0.693)	0.373	0.711	0.562	0.341	0.859	0.461
Model B: NLP^f^ free-text narratives	0.803 (0.792‐0.814)	0.621	0.704	0.751	0.474	0.890	0.567
Model C: multimodal fusion (baseline + NLP Narratives)	0.808 (0.798‐0.818)	0.630	0.720	0.738	0.571	0.845	0.637

a Performance metrics were evaluated on the independent 2025 temporal test cohort (n=10,191). The optimal classification threshold for metrics (sensitivity, specificity, PPV, NPV, *F*_1_-score) was determined using the Youden index exclusively on the training set and subsequently locked for application to the test cohort. Positive predictive value is inherently influenced by the lower outcome prevalence (24.2%) in the test set. The substantial improvement across all metrics, particularly the area under the receiver operating characteristic curve and *F*_1_-score, demonstrates the meaningful incremental prognostic value embedded within unstructured dispatch narratives.

b AUROC: area under the receiver operating characteristic curve.

c AUPRC: area under the precision-recall curve.

d PPV: positive predictive value.

e NPV: negative predictive value.

f NLP: natural language processing.

### Algorithm Robustness and Comparative Performance

**Figure 4.**
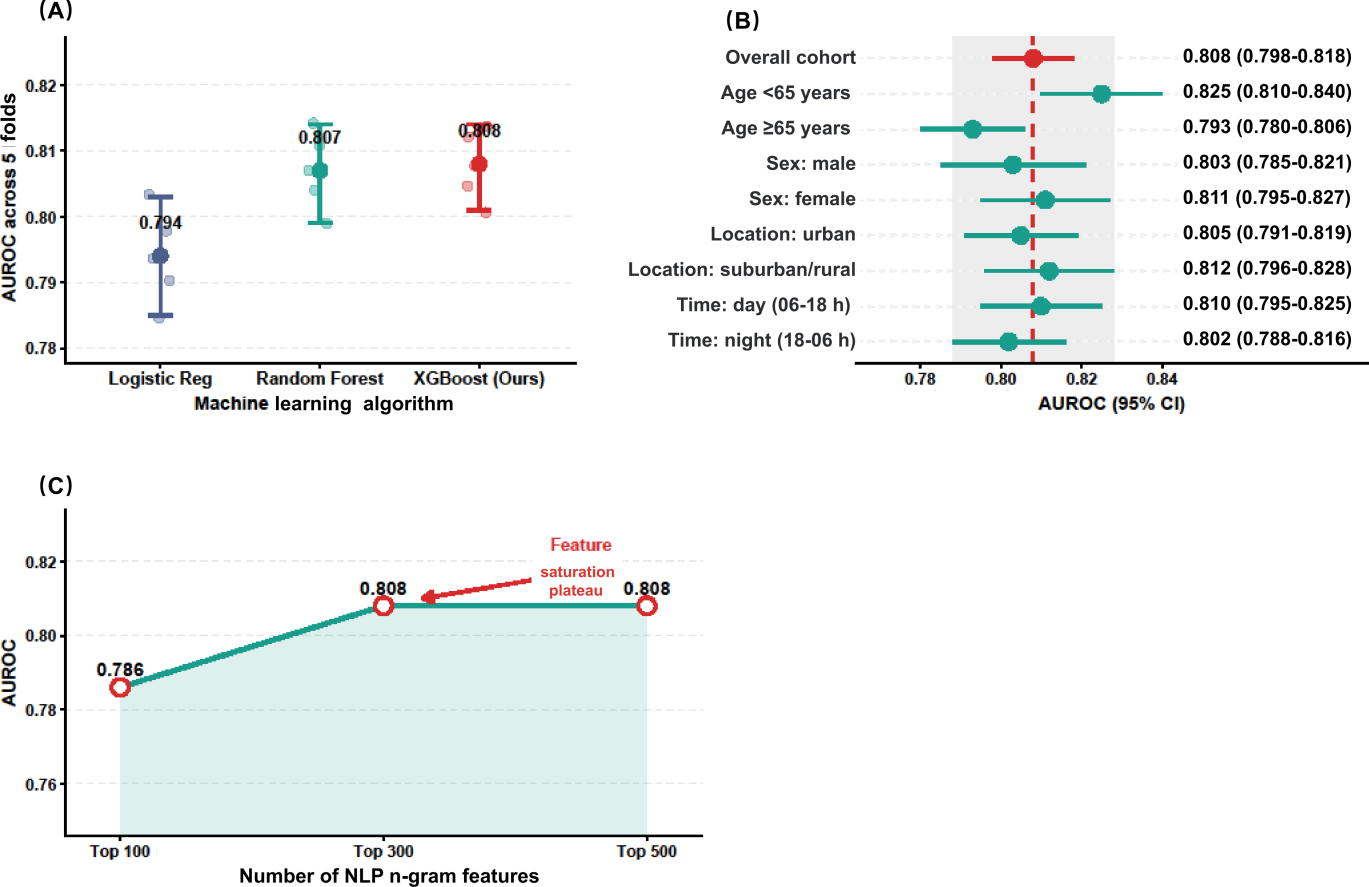
Algorithm robustness, subgroup fairness, and semantic feature saturation. (A) Jitter plot combined with point-ranges showing the stable area under the receiver operating characteristic curve (AUROC) variance across 5-fold cross-validation for penalized logistic regression, random forest, and the Extreme Gradient Boosting (XGBoost) model. (B) Forest plot demonstrating algorithmic fairness across key demographic and spatiotemporal subgroups in the test cohort. The vertical dashed red line represents the overall AUROC (0.808), and the gray shaded area indicates a ±2% tolerance margin. All subgroups fall within this margin, indicating unbiased predictive performance. (C) Area-line chart illustrating feature size stability. The predictive performance reached a saturation plateau at the top 300 NLP n-gram features, with no further incremental benefit observed upon expanding to 500 features. NLP: natural language processing.

Comparative evaluation across 3 machine learning algorithms—XGBoost, random forest, and penalized logistic regression—demonstrated consistent predictive performance (Figure 4A). The XGBoost model achieved the highest discrimination (AUROC 0.808), closely followed by the random forest model (AUROC 0.807), while penalized logistic regression yielded slightly lower performance (AUROC 0.794).

These similar performances across different modeling approaches suggest that the predictive signal primarily originated from the textual features themselves rather than from a specific algorithmic architecture. Subgroup analyses further demonstrated stable model performance across demographic and spatial groups (Figure 4B). The multimodal model achieved AUROC values of 0.825 in patients younger than 65 years and 0.793 in those aged 65 years or older. Performance was also comparable between male (AUROC 0.803) and female (AUROC 0.811) patients, as well as between urban (AUROC 0.805) and suburban or rural (AUROC 0.812) dispatch locations.

### Semantic Signatures of Criticality

**Figure 5.**
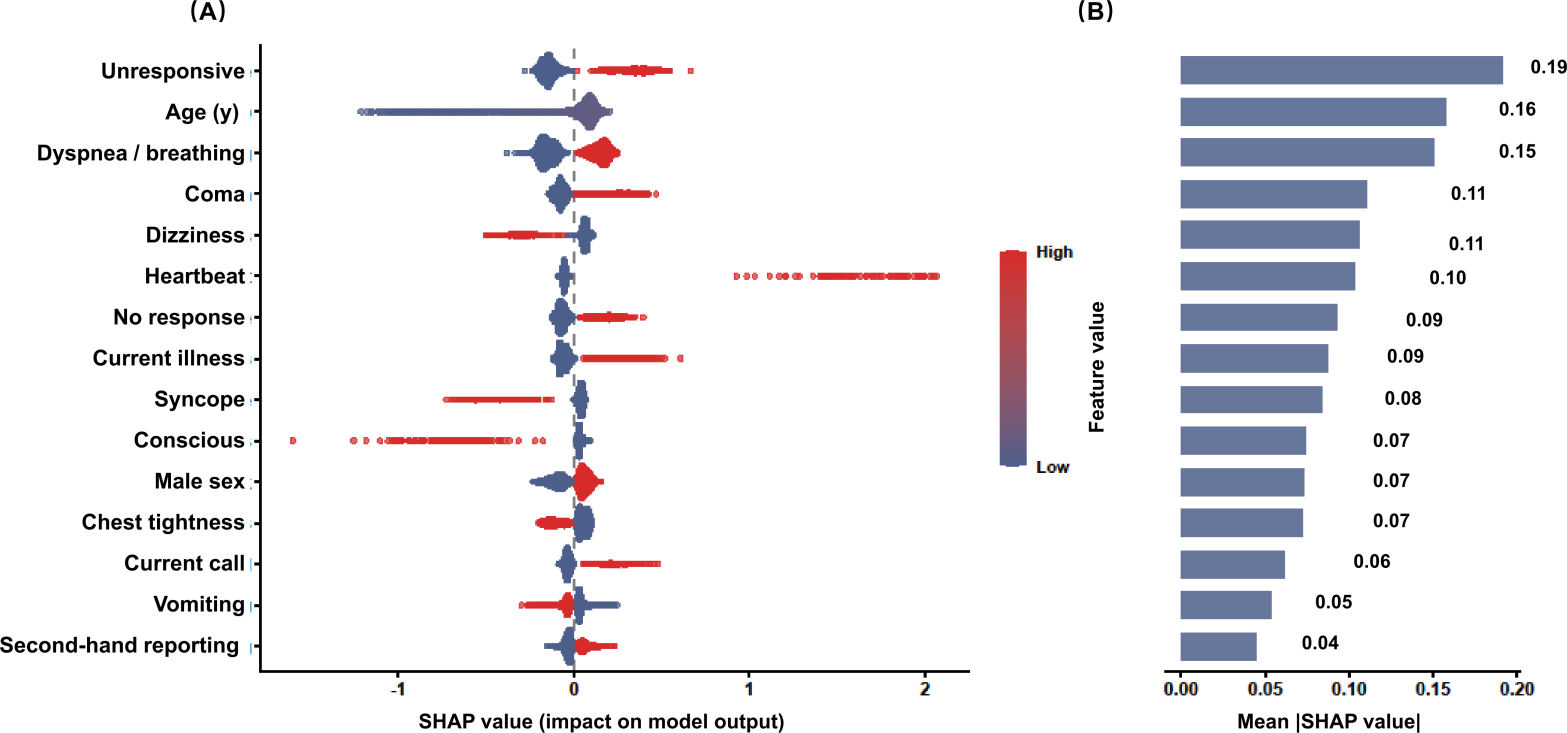
Semantic signatures of criticality derived from Shapley Additive Explanations (SHAP) analysis. (A) SHAP summary beeswarm plot illustrating the impact of the top 15 extracted features on the model’s output. Each dot represents an individual patient. The color gradient indicates the original feature value (red=high or present, blue=low or absent), while the position on the *x*-axis represents the SHAP value (positive values drive the prediction toward a critical outcome). Notably, implicit contextual cues, such as “Second-hand reporting” (symptoms reported by a bystander), were identified as high-risk indicators, alongside explicit symptoms such as “Unresponsive” and “Coma.” To enhance clinical interpretability, it is important to note that these isolated natural language processing (NLP) tokens represent longer dispatch phrases (eg, the high-risk feature “Heartbeat” typically corresponds to the caller stating “no heartbeat,” whereas the low-risk feature “Conscious” reflects “the patient is still conscious”). (B) Bar plot showing the mean absolute SHAP values, ranking the overall magnitude of each feature’s contribution to the automated triage decision.

Model interpretability was examined using SHAP analysis to identify the most influential textual features contributing to predictions. The SHAP summary plot revealed several clinically meaningful linguistic patterns associated with critical outcomes (Figure 5A).

Among the most influential features were terms describing impaired consciousness or respiratory distress, including “unresponsive,” “breathing,” “coma,” “no response,” “heartbeat,” and “dizziness.” Because isolated n-gram tokens can sometimes lack clinical context, reviewing the original source phrases provides better interpretability. For example, the high-risk token “heartbeat” predominantly appeared in dire phrases such as “has no heartbeat” or “heartbeat stopped.” Similarly, “breathing” was often extracted from urgent reports such as “can’t breathe” or “stopped breathing.” Conversely, features associated with lower risk, such as “conscious,” typically appeared in reassuring statements such as “the patient is still conscious.” These descriptors correspond to symptoms frequently reported by callers or bystanders when patients present with severe cardiopulmonary compromise. Notably, several influential features reflected second-hand descriptions from witnesses, suggesting that the inability of the patient to communicate directly may itself function as an important contextual signal of clinical severity. The semantic patterns identified by the model were consistent with established clinical indicators of critical illness and support the interpretability of the NLP-based approach (Figure 5B).

### Early-Warning Simulation and Clinical Usefulness

**Figure 6.**
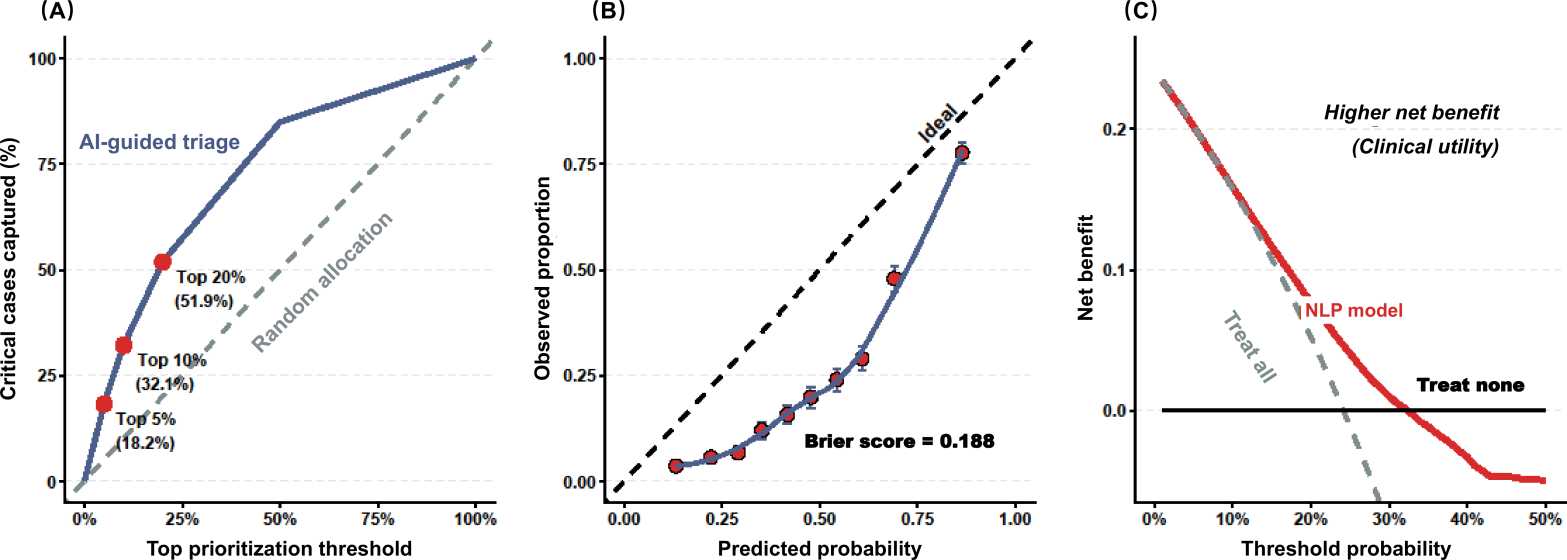
Early-warning clinical usefulness and decision curve analysis (DCA). (A) Early-warning risk enrichment curve demonstrating the proportion of true critical outcomes captured when prioritizing dispatch resources based on AI-predicted risk thresholds. Prioritizing the top 10% (n=1019) highest-risk calls successfully captured 32.1% (n=791) of all critical outcomes (a 3.2-fold risk enrichment compared to random allocation, dashed line). (B) Calibration plot assessing the agreement between predicted probabilities and observed critical outcome proportions across risk deciles. The blue error bars indicate 95% CIs for the observed proportions. The model yielded a Brier score of 0.188 (95% CI 0.185‐0.191). (C) DCA demonstrating that the natural language processing (NLP)–enabled multimodal model (red line) provides a higher net benefit than both the “treat-all” (gray dashed line) and “treat-none” (black solid line) strategies across all clinically relevant threshold probabilities for advanced life support (ALS) dispatch.

To evaluate the potential operational value of automated narrative-based triage, a risk enrichment analysis was conducted using the 2025 temporal validation cohort. When the model prioritized the top 10% (n=1019) of dispatch calls with the highest predicted risk scores, 32.1% (n=791) of all true critical outcomes were captured, representing a 3.2-fold enrichment compared with random allocation (Figure 6A).

Expanding the prioritization threshold to the top 20% (n=2038) of calls captured 51.9% (n=1279) of the critical outcomes, corresponding to a 2.6-fold enrichment. These findings suggest that even modest prioritization thresholds could substantially concentrate high-risk patients within a limited subset of EMS dispatch calls.

Based on the outcome prevalence of 24.2% in the temporal validation cohort, the null model yielded a Brier score of 0.183. The multimodal model achieved a Brier score of 0.188 (95% CI 0.185‐0.191, estimated via 1000 bootstrap resamples), with a calibration intercept of −1.27 and a calibration slope of 1.30. These findings indicate imperfect probability calibration (Figure 6B). The observed calibration deviation is likely attributable to the substantial prevalence shift between the development and temporal validation cohorts, together with the intentional class weighting strategy used during model development. Because the primary objective of this study was to evaluate temporal transportability and risk ranking of the original model, post hoc recalibration was not applied to the temporal validation cohort. Furthermore, DCA showed that the NLP-enabled multimodal model consistently provided greater net benefit than both treat-all and treat-none strategies across clinically relevant threshold probabilities (Figure 6C). Together, these results indicate that automated analysis of dispatch narratives may offer practical value for early risk stratification and optimized allocation of prehospital emergency resources.

## Discussion

### Principal Findings

In this population-based study of 38,523 EMS dispatches for suspected acute cardiopulmonary symptoms, we evaluated a narrative-based approach to early risk stratification using routinely collected dispatch texts. The findings suggest that information embedded in free-text narratives is associated with clinically relevant risk signals that are not captured by conventional structured variables. When incorporated into a multimodal framework, these semantic features were able to improve risk discrimination and remained relatively stable under temporal dataset shift. Rather than representing a stand-alone solution, these results indicate that narrative-derived features may serve as a complementary component within existing prehospital triage systems. Although the underlying NLP and machine-learning methods are well established, the primary contribution of this study lies in the rigorous temporal validation of this framework and in demonstrating its potential clinical applicability within a real-world EMS dispatch setting. These findings support the value of adapting established, interpretable machine learning methods to clinically relevant implementation challenges rather than introducing a novel prediction algorithm.

### Comparison With Prior Work

Early risk stratification is a central objective of prehospital care, particularly for acute cardiopulmonary presentations where delays in recognition may directly affect downstream management [15]. Existing dispatch systems largely rely on predefined symptom categories, structured checklists, or dispatcher experience. While such approaches provide operational consistency, they are inherently limited in capturing the complexity and evolution of acute illness prior to patient contact [16]. In parallel, there has been increasing interest in applying machine learning and NLP in clinical settings, including electronic health records, radiology reports, and clinical documentation, where unstructured text has been shown to contain meaningful prognostic information [17]. However, the use of narrative data at the dispatch level remains relatively underexplored, despite being the earliest available clinical description in the care pathway [18].

Previous work in EMS has primarily focused on structured data, such as response intervals, demographic characteristics, or predefined complaint codes, to support triage and system optimization [19]. Some studies have explored prediction models for cardiac arrest recognition or dispatch prioritization, but these approaches often rely on limited input features or are developed within static datasets [20,21]. In contrast, narrative-based approaches leverage the variability and richness of real-world communication to provide complementary prognostic perspectives on patient status.

The multimodal model remained superior even after strengthening the structured comparator by incorporating routinely available complaint categories, indicating that narrative-derived features provide clinically meaningful information beyond conventional structured dispatch variables. The observed association between narrative features and clinical outcomes likely reflects the way acute illness is described and perceived during emergency calls. Unlike structured variables, which represent relatively static patient characteristics (eg, age and sex), narrative descriptions capture dynamic information regarding symptom onset, progression, and contextual observations [22]. The modest incremental improvement achieved by multimodal integration suggests that narrative-derived features may already capture much of the clinically relevant information required for early risk stratification, thereby limiting the additional predictive contribution of basic structured variables when dispatch narratives are sufficiently informative. Nevertheless, structured variables may still provide complementary value in situations where narrative information is limited or incomplete, for example, because of communication difficulties or very brief emergency calls [23,24]. Taken together, these findings suggest that incorporating free-text dispatch narratives alongside structured information may improve early risk assessment and support more informed allocation of EMS resources. Crucially, transformer-based architectures have demonstrated excellent performance across a wide range of clinical NLP tasks by capturing complex contextual relationships. However, emergency dispatch narratives are typically short, fragmented, and highly abbreviated, and the incremental benefit of large language models in this setting remains uncertain. In addition, lightweight n-gram–based models offer greater interpretability through explicit feature attribution and generally require substantially fewer computational resources, which may facilitate future implementation in resource-constrained EMS dispatch environments. Future studies directly comparing lightweight and transformer-based approaches across diverse EMS datasets will be valuable to better define their respective strengths and optimal application scenarios [25,26].

From a clinical and operational perspective, the potential value of narrative-informed risk stratification lies in its timing [27]. Decisions regarding dispatch priority, resource allocation, and destination planning are often made before direct patient assessment. In this context, even modest improvements in early risk identification may influence preparedness at multiple levels, including equipment readiness, crew configuration, and prenotification of receiving facilities [28]. Rather than replacing existing triage protocols, a narrative-based risk signal could be integrated as an additional layer of information to support decision-making under uncertainty [29]. This may be particularly relevant in settings with constrained ALS resources, where aligning resource intensity with patient risk remains a persistent challenge [30].

### Limitations

Several limitations should be considered when interpreting these findings. First, the analysis depends on the accuracy and completeness of dispatch narratives, which may vary according to dispatcher training, caller communication ability, and situational stress. Misclassification or information loss at the transcription stage cannot be excluded. Second, although temporal validation was performed, the data were derived from a single EMS system in China. Differences in language, dispatch workflows, documentation practices, and health care system organization may limit the applicability of the proposed model to other regions and countries. Third, the outcome definition is based on prehospital assessments and does not capture in-hospital end points such as definitive diagnosis, survival, or neurological recovery. Furthermore, our composite “critical outcome” relies on local EMS operational criteria and intervention thresholds. Although the use of standardized structured fields likely improves internal consistency within our system, these operational practices may vary across different regional EMS networks, potentially affecting the external generalizability of our outcome definition. However, from an EMS operational perspective, the occurrence of a prehospital critical outcome represents the most direct and actionable proxy for dispatch triage because it directly informs decisions regarding on-scene ALS and transport prioritization, independent of downstream in-hospital management. Fourth, our NLP pipeline relied on character-level n-grams and tree-based ensemble models. Although these methods offer advantages in interpretability and computational efficiency, they may not fully capture complex semantic relationships compared with modern pretrained language models. Finally, although the lightweight architecture suggests favorable computational efficiency, deployment-oriented characteristics were not formally evaluated in this retrospective study. Therefore, our findings support the potential for future implementation rather than confirmed operational deployment. Future multicenter studies incorporating external validation, prospective workflow evaluation, and implementation-oriented engineering assessments will be essential to determine the feasibility of integrating this framework into routine EMS dispatch practice.

### Conclusions

Free-text dispatch narratives contain clinically relevant information that is associated with early risk stratification in patients with suspected cardiopulmonary emergencies. Incorporating these narrative features into a structured modeling framework may provide additional insight beyond conventional variables and support decision-making at the dispatch stage. Further validation and prospective evaluation are needed to determine how such approaches can be integrated into routine prehospital care. These findings support the potential value of incorporating explainable, NLP-derived information into EMS dispatch systems, providing a foundation for future multicenter validation and prospective implementation studies.

## Supplementary material

10.2196/97672 Multimedia Appendix 1 Methodological details and model specifications.

10.2196/97672 Checklist 1 TRIPOD checklist.

## Abbreviations

ALS: advanced life support
AUPRC: area under the precision-recall curve
AUROC: area under the receiver operating characteristic curve
BERT: bidirectional encoder representations from transformers
DCA: decision curve analysis
EMS: emergency medical services
NLP: natural language processing
PPV: positive predictive value
SHAP: Shapley Additive Explanations
SpO_2_: oxygen saturation
TF-IDF: frequency-inverse document frequency
TRIPOD: Transparent Reporting of a Multivariable Prediction Model for Individual Prognosis or Diagnosis
XGBoost: Extreme Gradient Boosting
