# Supplementary material for: Predicting Critical Outcomes in Suspected Cardiopulmonary Emergencies Using Dispatch Narratives: Temporal Validation Study
Source: J Med Internet Res. 2026 Aug 14;28:e97672. doi: 10.2196/97672 (PMC13476006; doi:10.2196/97672)
Supplement: Supplementary file 1 [file jmir-v28-e97672-s002.pdf]

TRIPOD Checklist: Prediction Model Development and Validation

Item	Section/Topic	Description	Reported in Section / Paragraph
**Title and abstract**			
1	Title	Identify the study as developing and/or validating a multivariable prediction model, the target population, and outcomes to be predicted.	Title
2	Abstract	Provide a summary of objectives, study design, setting, participants, sample size, predictors, outcome, statistical analysis, results, and conclusions.	Abstract
**Introduction**			
3a	Background	Explain the medical context (including whether diagnostic or prognostic) and rationale for developing or validating the multivariable prediction model, including references to existing models.	Section 1 (Introduction), Paragraphs 1-3
3b	Objectives	Specify the objectives, including whether the study describes the development or validation of the model or both.	Section 1 (Introduction), Paragraph 4
**Methods**			
4a	Source of data	Describe the study design or source of data (e.g., randomized trial, cohort, or registry data), separately for the development and validation data sets, if applicable.	Section 2.1
4b	Source of data	Specify the key study dates, including start of accrual; end of	Section 2.2

		accrual; and, if applicable, end of follow-up.	
5a	Participants	Specify key elements of the study setting (e.g., primary care, secondary care, general population) including number and location of centres. Describe eligibility criteria for participants.	Section 2.1 & 2.2
5b	Participants	Where applicable, describe treatments received.	N/A (Prehospital triage stage)
5c	Participants	Where applicable, describe details of the study follow-up.	N/A (Immediate operational outcome)
6a	Outcome	Clearly define the outcome that is predicted by the prediction model, including how and when assessed.	Section 2.3
6b	Outcome	Report any actions to blind assessment of the outcome to be predicted.	Section 2.3 (Objective logic checks used)
7a	Predictors	Clearly define all predictors used in developing or validating the multivariable prediction model, including how and when they were measured.	Section 2.4 & 2.5
7b	Predictors	Report any actions to blind assessment of predictors for the outcome and other predictors.	Section 2.4 (Temporal isolation)
8	Sample size	Explain how the study size was arrived at.	Section 2.2
9	Missing data	Describe how missing data were handled (e.g., complete-case analysis, single imputation, multiple imputation) with details of any imputation method.	Section 2.2
10a	Statistical analysis	Describe how predictors were handled in the analyses.	Section 2.4 & 2.5

10b	Statistical analysis	Specify type of model, all model-building procedures (including any predictor selection), and method for internal validation.	Section 2.5
10c	Statistical analysis	For validation, describe how the predictions were calculated.	Section 2.6
10d	Statistical analysis	Specify all measures used to assess model performance and, if relevant, to compare multiple models.	Section 2.6 & 2.7
10e	Statistical analysis	Describe any model updating (e.g., recalibration) arising from the validation, if done.	N/A
11	Risk groups	Provide details on how risk groups were created, if done.	Section 2.6 & 3.5
12	Development vs. validation	For validation, identify any differences from the development data in setting, eligibility criteria, outcome, and predictors.	N/A (Same setting, temporal validation)
**Results**			
13a	Participants	Describe the flow of participants through the study, including the number of participants with and without the outcome and, if applicable, a summary of the follow-up time. A diagram may be helpful.	Section 3.1 & Figure 1
13b	Participants	Describe the characteristics of the participants (basic demographics, clinical features, available predictors), including the number of participants with missing data for predictors and outcome.	Section 3.1 & Table 1
13c	Participants	For validation, show a comparison with the development data of the	Section 3.1 & Table 1

		distribution of important variables (demographics, predictors and outcome).	
14a	Model development	Specify the number of participants and outcome events in each analysis.	Section 3.1
14b	Model development	If done, report the unadjusted association between each candidate predictor and outcome.	N/A (NLP high-dimensional)
15a	Model specification	Present the full prediction model to allow predictions for individuals (i.e., all regression coefficients, and model intercept or baseline survival at a given time point).	Section 3.4 & Figure 5 (SHAP)
15b	Model specification	Explain how to use the prediction model.	Section 3.5
16	Model performance	Report performance measures (with CI) for the prediction model.	Sections 3.2, 3.3, Table 2 & Figs 3, 6
17	Model updating	If done, report the results from any model updating (i.e., model specification, model performance).	N/A
**Discussion**			
18	Limitations	Discuss any limitations of the study (such as nonrepresentative sample, few events per predictor, missing data).	Discussion (Limitations)
19a	Interpretation	For validation, discuss the results with reference to performance in the development data, and any other validation data.	Discussion (Comparison with Prior Work)
19b	Interpretation	Give an overall interpretation of the results, considering objectives, limitations, results from similar studies, and other relevant evidence.	Discussion (Principal Findings)

20	Implications	Discuss the potential clinical use of the model and implications for future research.	Section 5 (Conclusions)
**Other information**			
21	Supplementary info	Provide information about the availability of supplementary resources, such as study protocol, Web calculator, and data sets.	Declarations (Data availability)
22	Funding	Give the source of funding and the role of the funders for the present study.	Declarations (Funding)
