# Supplementary material for: Predicting Critical Outcomes in Suspected Cardiopulmonary Emergencies Using Dispatch Narratives: Temporal Validation Study
Source: J Med Internet Res. 2026 Aug 14;28:e97672. doi: 10.2196/97672 (PMC13476006; doi:10.2196/97672)
Supplement: Supplementary file 2 [file jmir-v28-e97672-s001.docx]

**Supplementary Appendix 1: Methodological Details and Model Specifications**

**1. Exact Outcome Coding Rules**
The composite "critical outcome" was strictly defined using objective operational criteria documented in the prehospital EMS records:
(1) Documented death at the scene;
(2) Persistent hemodynamic or respiratory instability despite initial resuscitative efforts (e.g., systolic blood pressure persistently < 90 mmHg requiring vasopressors, or peripheral capillary oxygen saturation [SpO2] < 90% despite high-flow oxygen);
(3) Requirement for immediate advanced life support (ALS) interventions.

**2. Natural Language Processing (NLP) Resources**
The NLP pipeline processed Chinese dispatch narratives using a character-level n-gram (specifically bigrams) tokenization strategy. Feature extraction was constrained to the top 300 most frequent n-grams derived exclusively from the training corpus to prevent information leakage.

**3. Hyperparameter Tuning**
Hyperparameters for the extreme gradient boosting (XGBoost) model were optimized during model development using cross-validation on the training cohort. Parameters considered during model development included learning rate, maximum tree depth, number of boosting rounds, subsampling ratio, and column sampling ratio.

Because the final optimized parameter set was not retained in the exported training workspace, the exact parameter combination cannot be retrospectively reported. The complete model development workflow, feature engineering procedure, and validation strategy are fully described in the Methods to facilitate methodological replication.

**4. Classification Threshold Strategy**
Predicted probabilities were retained as continuous outputs for all primary model evaluations, including discrimination (AUROC, AUPRC), calibration, and decision curve analysis. Binary thresholds were applied only when threshold-dependent performance measures were calculated.
